# Topological gapless phase in Kitaev model on square lattice

**DOI:** 10.1038/s41598-017-17334-w

**Published:** 2017-12-07

**Authors:** P. Wang, S. Lin, G. Zhang, Z. Song

**Affiliations:** 1 School of Physics, Nankai University, Tianjin, 300071 China; 2 Science and Technology on Electro-Optical Information Security Control Laboratory, Tianjin, 300308 China; 3 College of Physics and Materials Science, Tianjin Normal University, Tianjin, 300387 China

## Abstract

We study the topological feature of gapless states in the fermionic Kitaev model on a square lattice. There are two types of gapless states which are topologically trivial and nontrivial. We show that the topological gapless phase lives in a wide two-dimensional parameter region and are characterized by two vertices of an auxiliary vector field de-fined in the two-dimensional momentum space, with opposite winding numbers. The isolated band touching points, as the topological defects of the field, move, emerge, and disappear as the parameters vary. The band gap starts to open only at the merg-ing points, associated with topologically trivial gapless states. The symmetry protect-ing the topological gapless phase and the robustness under perturbations are also discussed.

## Introduction

The concept of topology in condensed matter physics has received great attention^1–4^ as it not only predicts new physical phenomena with potential technological applications, but is also closely related to fundamental physics, such as the discovery of fermionic particles and phenomena predicted in high-energy physics, including Majorana^5–10^, Dirac^11–17^ and Weyl fermions^18–26^. These concepts relate to an interesting topic, topological gapless phase. System in the topological gapless phase exhibits band structures with band-touching points in the momentum space, where these kinds of nodal points appear as topological defects of an auxiliary vector field. Then these points are unremovable due to the symmetry protection, until a pair of them meets and annihilates together. On the other hand, a gapful phase can be topologically non-trivial, commonly referred to as topological insulators and superconductors, the band structure of which is characterized by nontrivial topology.

In this paper, we study the topological gapless phase in the Kitaev model on a square lattice based on analytical solutions. It has been shown that a large class of two-dimensional spinless fermion models exhibit topological superconducting phases^27^. Two different phases are separated by the gapless state as quantum phase boundary. We will show that there are two types of gapless states which are topologically trivial and nontrivial. The topological gapless states are characterized by two vertices with opposite winding numbers in the two-dimensional momentum space. As parameters vary, the isolated topological band touching points, as topological defects, move, emerge, and disappear as the band gap opens. In general, a gapless state only lives at a boundary line, which separates two gapful phases. We show that the topological gapless phase in the present model exist in a wide two-dimensional parameter region. We also analyze the symmetry which protects the topological gapless states the robustness under perturbations. In contrast to previous study, this symmetry does not involve any anti-unitary operation.

## Model and phase diagram

We consider the Kitaev model on a square lattice which is employed to depict 2D *p*-wave superconductors. The Hamiltonian of the tight-binding model on a square lattice takes the following forma

1 $$\begin{array}{rcl}H & = & -t\sum _{{\bf{r}},{\bf{a}}}{c}_{{\bf{r}}}^{\dagger }{c}_{{\bf{r}}+{\bf{a}}}-{\rm{\Delta }}\sum _{{\bf{r}},{\bf{a}}}{c}_{{\bf{r}}}{c}_{{\bf{r}}+{\bf{a}}}+{\rm{h}}{\rm{.c}}.\\  &  & +\mu \sum _{{\bf{r}}}(2{c}_{{\bf{r}}}^{\dagger }{c}_{{\bf{r}}}-1),\end{array}$$

where **r** is the coordinates of lattice sites and ***c***_**r**_ is the fermion annihilation operators at site **r**. Vectors **a** = *a* **i**, *a* **j**, are the lattice vectors in the *x* and *y* directions with unitary vectors **i** and **j**. The hopping between (pair operator of) neighboring sites is described by the hopping amplitude *t* (the real order parameter Δ). The last term gives the chemical potential.

Imposing periodic boundary conditions on both directions, the Hamiltonian can be exactly diagonalized. Taking the Fourier transformation

2 $${c}_{{\bf{r}}}=\frac{1}{N}\sum _{{\bf{k}}}{c}_{{\bf{k}}}{e}^{i{\bf{k}}\cdot {\bf{r}}},$$

we get

3 $$\begin{array}{rcl}H & = & \sum _{{\bf{k}}}[2(\mu -t\,\cos \,{k}_{x}-t\,\cos \,{k}_{y}){c}_{{\bf{k}}}^{\dagger }{c}_{{\bf{k}}}\\  &  & -i{\rm{\Delta }}(\sin \,{k}_{y}+\,\sin \,{k}_{x})({c}_{{\bf{k}}}^{\dagger }{c}_{-{\bf{k}}}^{\dagger }+{c}_{{\bf{k}}}{c}_{-{\bf{k}}})-\mu ],\end{array}$$

where the summation of **k** = (*k*_*x*_, *k*_*y*_) is ${\sum }_{{\bf{k}}}={\sum }_{{k}_{x}=-\pi }^{\pi }{\sum }_{{k}_{y}=-\pi }^{\pi }$. The Hamiltonian can be diagonalized as

4 $$H=\sum _{{\bf{k}}}{\varepsilon }_{{\bf{k}}}({\gamma }_{{\bf{k}}}^{\dagger }{\gamma }_{{\bf{k}}}-\frac{1}{2}),$$

by introducing the Bogoliubov operator

5 $${\gamma }_{{\bf{k}}}={u}_{{\bf{k}}}{c}_{{\bf{k}}}+{v}_{{\bf{k}}}{c}_{-{\bf{k}}}^{\dagger },$$

which satisfies the commutation relations of fermion

6 $$\{{\gamma }_{{\bf{k}}},{\gamma }_{{\bf{q}}}^{\dagger }\}={\delta }_{{\bf{k}},{\bf{q}}},\{{\gamma }_{{\bf{k}}},{\gamma }_{{\bf{q}}}\}=\{{\gamma }_{{\bf{k}}}^{\dagger },{\gamma }_{{\bf{q}}}^{\dagger }\}=0.$$

Here the spectrum is

7 $${\varepsilon }_{{\bf{k}}}=2\sqrt{{[t(\cos {k}_{x}+\cos {k}_{y})-\mu ]}^{2}+{{\rm{\Delta }}}^{2}{(\sin {k}_{x}+\sin {k}_{y})}^{2}}$$

and the normalized amplitudes are

8 $${u}_{{\bf{k}}}=i\,\sin \,\frac{{\theta }_{{\bf{k}}}}{2},{v}_{{\bf{k}}}=\,\cos \,\frac{{\theta }_{{\bf{k}}}}{2},$$

where *θ*_k_ is determined by

9 $$\tan \,{\theta }_{{\bf{k}}}=\frac{{\rm{\Delta }}(\sin \,{k}_{y}+\,\sin \,{k}_{x})}{t(\cos \,{k}_{x}+\,\cos \,{k}_{y})-\mu }.$$

The ground state can be constructed as

10 $$|{\rm{G}}\rangle =\prod _{{\bf{k}}}{\gamma }_{{\bf{k}}}|{\rm{Vac}}\rangle ,$$

with the groundstate energy

11 $${E}_{{\rm{g}}}=-\frac{1}{2}\sum _{{\bf{k}}}{\varepsilon }_{{\bf{k}}},$$

where $|{\rm{Vac}}\rangle$ is the vacuum state of *c*_k_, satisfying ${c}_{{\bf{k}}}|{\rm{Vac}}\rangle =0$ for all **k**. We note that the gapless ground state appears when *ε*_k_ has a zero point, or band touching point of single *γ*_k_-particle spectrum.

In this paper, we are interested in the gapless state arising from the band touching point of the spectrum. The band degenerate point **k**_0_ = (*k*_0*x*_, *k*_0*y*_) is determined by

12 $$\{\begin{array}{l}{\rm{\Delta }}(\sin \,{k}_{0x}+\,\sin \,{k}_{0y})=0,\\ \mu -t(\cos \,{k}_{0x}+\,\cos \,{k}_{0y})=0.\end{array}$$

For different parameters, two bands touch at three types of configurations, single point, double points, and curves in the *k*_*x*_-*k*_*y*_ plane. There are two typical cases:

(i) For Δ ≠ 0, we have

13 $${k}_{0x}=-{k}_{0y}=\pm \arccos (\frac{\mu }{2t})$$

in the region $|\mu /t|\le 2$. It indicates that there are two nodal points for *μ* ≠ 0 and |*μ*/*t*| ≠ 2. The two points move along the line represented by the equation *k*_0*x*_ = − *k*_0*y*_, and merge at **k**_0_ = (π, −π) when *μ*/*t* = ±2. In the case of *μ* = 0, the nodal points become two nodal lines represented by the equations *k*_0y_ = ±π + *k*_0*x*_.

(ii) For Δ = 0, the system becomes interaction free. In this case, Eq. (12) becomes

14 $${k}_{0y}=\pm \arccos \,(\frac{\mu }{t}-\,\cos \,{k}_{0x})$$

in the region

15 $$0 < |\frac{\mu }{t}| < 2.$$

which indicates that the band gap close at the nodal line represented by Eq. (14). In the case of |*μ*/*t*| = 0, Eq. (12) represents four lines

16 $${k}_{0y}=\pm {k}_{0x}\pm \pi ,$$

and

17 $${k}_{0y}=\pm {k}_{0x}\mp \pi \mathrm{.}$$

In the case of |*μ*/*t*| = 2, the nodal points reduce to a single point.

**Figure 1:**
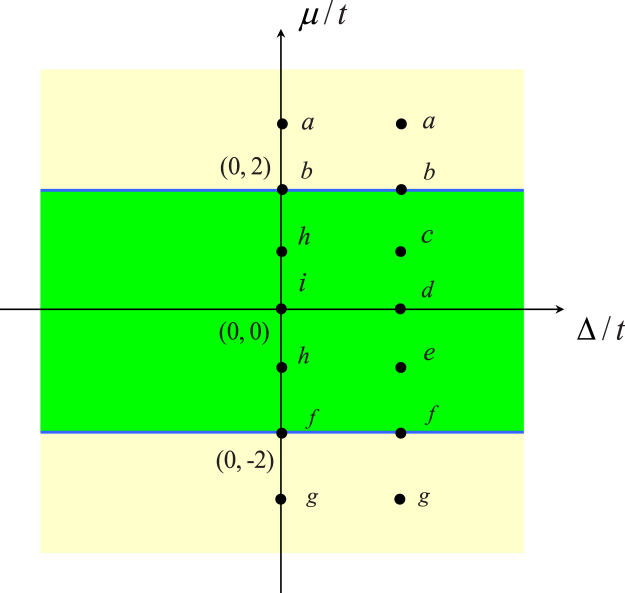
Phase diagram of the Kitaev model on a square lattice system on the parameter *μ* − Δ plane (in units of *t*). The blue lines indicate the boundary, which separate the gapful phases (yellow) and gapless phase (green). Several points (**a**–**g**) at typical positions are indicated and the same letter represents the situations with the similar band structures. The corresponding band structures and the topology of the nodal point in the momentum space are given in Figs 2 and 3, respectively.

**Figure 2:**
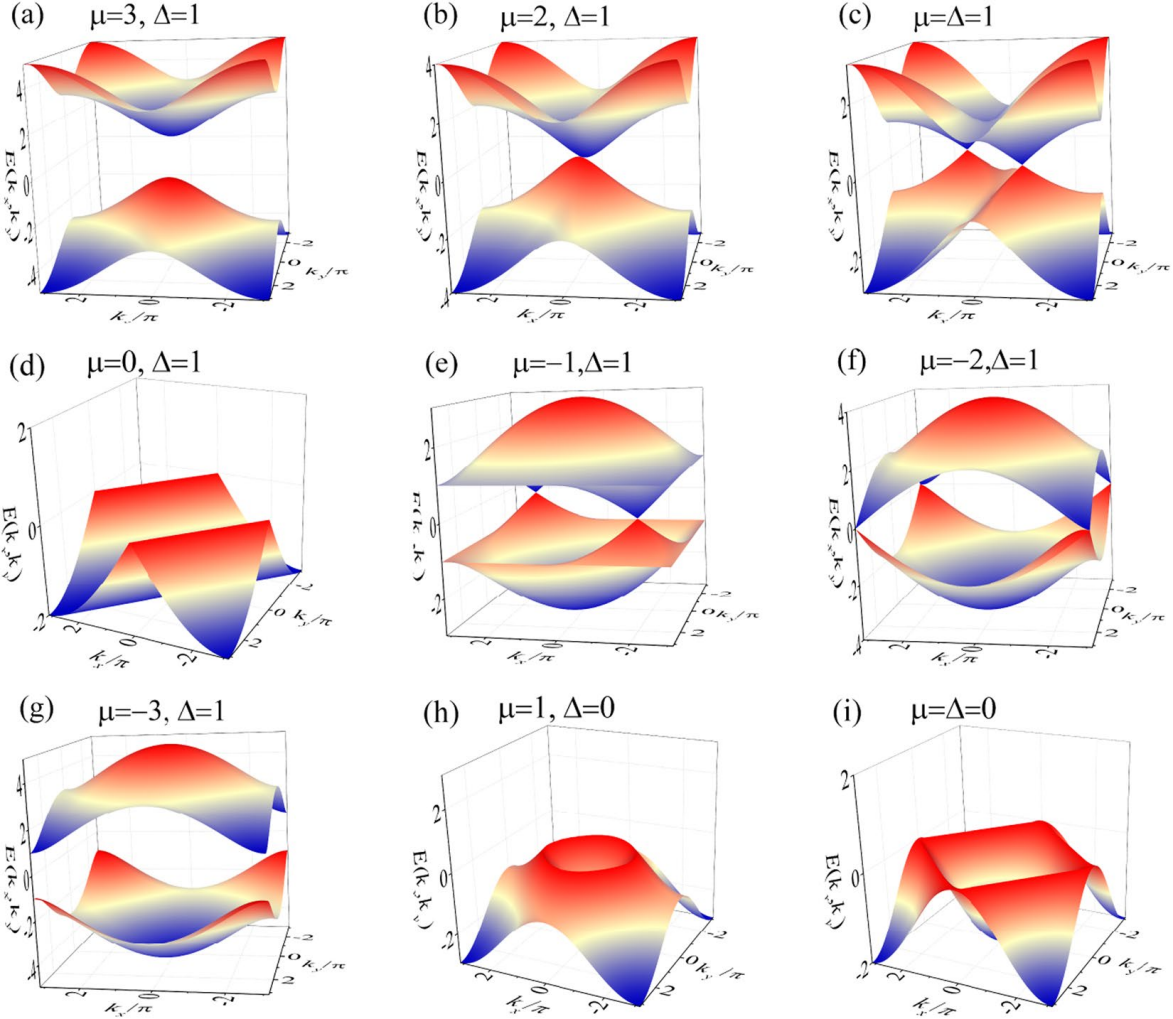
Energy spectra from Eq. (7) at points (**a**–**g**) marked in the phase diagram in Fig. 1. We see that (i) in the gapless phase region with Δ*μ* ≠ 0 (**b**,**c**,**e**,**f**), the zero energy points are isolated points; (ii) in the gapless phase region with Δ*μ* = 0 (**d**,**h**,**i**), the zero energy points become lines; (iii) At the phase boundary (**b**,**f**), two isolated points merge; and (iv) the zero energy points disappear in the gapful regions (**a**,**g**). In the cases of (**d**,**h**,**i**), we only plot the lower bands in order to display the entire nodal lines. In the momentum plane, both *k*_*x*_ and *k*_*y*_ range from −*π* to *π*.

The phase diagram is illustrated in Fig. 1, depending on the values of *μ* and Δ (compared with the hopping strength *t*). We select several points in the phase diagram of Fig. 1, which are representative of all possible typical cases. In addition, we plot the band structures in Fig. 2 for these cases. The configurations of nodal points in the figures are in agreement with our analysis.

## Topological nodal points

In this section, we will show that zero-gap systems are in the topological gapless phase. We demonstrate this point by rewriting the Hamiltonian in the form

18 $$H=\sum _{{\bf{k}}}(\begin{array}{cc}{c}_{{\bf{k}}}, & {c}_{-{\bf{k}}}^{\dagger }\end{array}){h}_{{\bf{k}}}(\begin{array}{c}{c}_{{\bf{k}}}^{\dagger }\\ {c}_{-{\bf{k}}}\end{array}),$$

where

19 $${h}_{{\bf{k}}}=(\begin{array}{cc}-\mu +t\,\cos \,{k}_{x}+t\,\cos \,{k}_{y} & -i{\rm{\Delta }}(\sin \,{k}_{y}+\,\sin \,{k}_{x})\\ i{\rm{\Delta }}(\sin \,{k}_{y}+\,\sin \,{k}_{x}) & \mu -t\,\cos \,{k}_{x}-t\,\cos \,{k}_{y}\end{array})\mathrm{.}$$

The core matrix can be expressed as

20 $${h}_{{\bf{k}}}={\bf{B}}({\bf{k}})\cdot {{\boldsymbol{\sigma }}}_{{\bf{k}}},$$

where the components of the auxiliary field ***B***(**k**) = (*B*_*x*_, *B*_*y*_, *B*_*z*_) are

21 $$\{\begin{array}{l}{B}_{x}={\rm{\Delta }}(\sin \,{k}_{y}+\,\sin \,{k}_{x})\\ {B}_{y}=-\mu +t(\cos \,{k}_{x}+\,\cos \,{k}_{y})\\ {B}_{z}=0\end{array}.$$

The Pauli matrices **σ**_**k**_ are taken as the form

22 $${\sigma }_{x}=(\begin{array}{cc}0 & -i\\ i & 0\end{array}),{\sigma }_{y}=(\begin{array}{cc}1 & 0\\ 0 & -1\end{array}),{\sigma }_{z}=(\begin{array}{cc}0 & 1\\ 1 & 0\end{array})\mathrm{.}$$

In condensed matter physics, the Dirac or Weyl point acts like a singularity of the Berry curvature in the Brillouin zone, or a magnetic monopole in *k*-space. When a degenerate point is isolated, it should be a vortex of the vector field **B**(**k**), which is the topological defect of the field. We will show that the appearance of gapless states in the present model and their existence regions are fully determined by the topological configurations of the field defects. And these defects can be classified as topologically trivial and non-trivial using topological number, or invariant. The topological invariant of a defect is the winding number

23 $$w=\frac{1}{2\pi }{\oint }_{C}d{\bf{k}}({\hat{B}}_{y}\nabla {\hat{B}}_{x}-{\hat{B}}_{x}\nabla {\hat{B}}_{y}),$$

where the unit vector $\hat{B}({\bf{k}})={\bf{B}}({\bf{k}})/|{\bf{B}}({\bf{k}})|$ and ∇ = ∂/∂***k*** is the nabla operator in *k*-space. On the other hand, the winding number of the vortices (the isolated degenerate points) is equivalent to the chirality of Weyl fermion in the context of quantum field theory.

Actually, in the vicinity of the defects, we have

24 $$\{\begin{array}{l}{B}_{x}={\rm{\Delta }}\,\cos \,{k}_{0x}({q}_{y}+{q}_{x})\\ {B}_{y}=t\,\sin \,{k}_{0x}({q}_{y}-{q}_{x})\\ {B}_{z}=0\end{array},$$

where **q** = **k** − **k**_0_, **k**_0_ = (*k*_0*x*_, *k*_0*y*_) and (*k*_0*x*_, *k*_0*y*_) satisfy Eq. (13), is the momentum in another frame. Around these degenerate points, the Hamiltonian *h*_**k**_ can be linearized as the form

25 $$ {\mathcal H} ({\bf{q}})=\sum _{i,j}{a}_{ij}{q}_{i}{\sigma }_{j},$$

which is equivalent to the Hamiltonian for two-dimensional massless relativistic fermions. The corresponding chirality for these particle is defined as

26 $$w={\rm{sgn}}[{\rm{\det }}({a}_{ij}\mathrm{)].}$$

**Figure 3:**
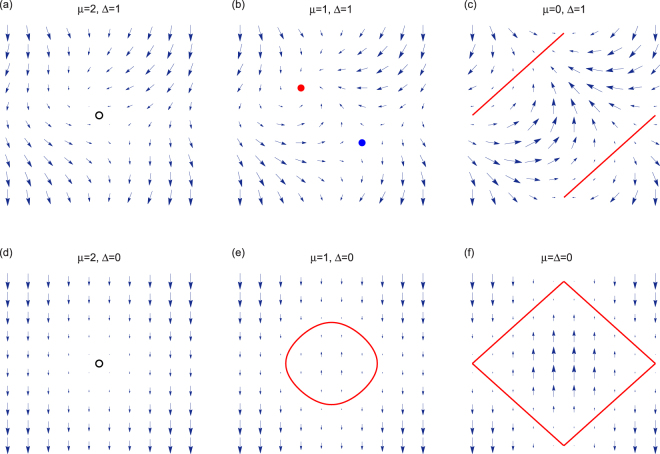
Isolated zero energy points as topological defects. The planar vector fields defined in Eq. (24) in the momentum space for several typical cases indicated in Fig. (1). It shows that the isolated degeneracy points correspond to the vortices in the momentum space with winding numbers 0 and ±1. The red point represents winding number −1 and the bule one 1 in Fig. (3b). The nontrivial winding number indicates that the isolated nodal point is topologically protected and thus cannot be removed in adiabatic procedures. Two zero energy points (red and blue dots) can merge to a single vortex (black empty circle) with zero winding number. Red lines denote the nodal lines. Abscissa and ordinate represent *k*_*x*_ and *k*_*y*_ respectively, ranging from −*π* to *π*.

Then we have

27 $${\rm{\det }}|\begin{array}{cc}{\rm{\Delta }}\,\cos \,{k}_{0x} & -t\,\sin \,{k}_{0x}\\ {\rm{\Delta }}\,\cos \,{k}_{0x} & t\,\sin \,{k}_{0x}\end{array}|=t{\rm{\Delta }}\,\sin \,2{k}_{0x},$$

which leads to *w* = ±1 for two nodal points. The chiral relativistic fermions serve as two-dimensional Weyl fermions. Two Weyl nodes located at two separated degenerate points have opposite chirality. We note that for Δ|*μ*/*t*|(|*μ*/*t*| − 2) = 0, we have *w* = 0. At this situation, two Weyl nodes merge at (0, 0) and (±π, ∓π). The topology of the nodal point becomes trivial, and a perturbation hence can open up the energy gap. We illustrate the vortex structure of the degeneracy point in *k*_*x*_-*k*_*y*_ plane in Fig. 3. As shown in figures, we find three types of topological configurations: pair of vortices with opposite chirality, single trivial vortex (or degeneracy lines), and no vortex, corresponding to topological gapless, trivial gapless and gapped phases, respectively.

## Symmetry protection of nodal points

In this section, we focus on the symmetry, which protects the nodal point or gapless state. We begin with the translational symmetry of the system. The Hamiltonian is invariant via a translational transformation, i.e., [*T*_*x*_, *H*] = 0 and [*T*_*y*_, *H*] = 0, where *T*_*x*_ and *T*_*y*_ are the shift operators defined as

28 $${\hat{T}}_{x}{c}_{{\bf{r}}}{\hat{T}}_{x}^{-1}={c}_{{\bf{r}}+{\bf{i}}},{\hat{T}}_{y}{c}_{{\bf{r}}}{\hat{T}}_{y}^{-1}={c}_{{\bf{r}}+{\bf{j}}}.$$

In *k*-space, the actions of the operators are

29 $${T}_{x}{c}_{{\bf{k}}}{T}_{x}^{-1}={c}_{{\bf{k}}}{e}^{i{k}_{x}},{T}_{y}{c}_{{\bf{k}}}{T}_{y}^{-1}={c}_{{\bf{k}}}{e}^{i{k}_{y}}.$$

Combining with the above two operators we have

30 $$[H,{\hat{T}}_{x-y}]=0,$$

where ${\hat{T}}_{x-y}={\hat{T}}_{x}-{\hat{T}}_{y}$. In general, for an excited state $|\psi ({k}_{x},{k}_{y})\rangle ={\gamma }_{{\bf{k}}}^{\dagger }|{\rm{G}}\rangle$, which is also the eigenstate of operators ${\hat{T}}_{x}$, ${\hat{T}}_{y}$, and ${\hat{T}}_{x-y}$, we have

31 $${\hat{T}}_{x}|\psi ({k}_{x},{k}_{y})\rangle ={e}^{i{k}_{x}}|\psi ({k}_{x},{k}_{y})\rangle ,$$

32 $${\hat{T}}_{y}|\psi ({k}_{x},{k}_{y})\rangle ={e}^{i{k}_{y}}|\psi ({k}_{x},{k}_{y})\rangle .$$

The two-fold zero-energy degenerate states can be constructed as

33 $$|{\psi }_{0}^{\pm }\rangle =\frac{1}{\sqrt{2}}(|\psi (-{k}_{0},{k}_{0})\rangle \pm |\psi ({k}_{0},-{k}_{0})\rangle ),$$

where *k*_0_ = |*k*_0*x*_|. It is easy to check that $|{\psi }_{0}^{\pm }\rangle$ is not the eigenstate of ${\hat{T}}_{x-y}$, and

34 $${\hat{T}}_{x-y}|{\psi }_{0}^{\pm }\rangle =-2i\,\sin \,{k}_{0}|{\psi }_{0}^{\mp }\rangle .$$

This indicates that the nodal points are protected by the symmetry related to ${\hat{T}}_{x-y}$. Furthermore, the zero-energy excited state $|{\psi }_{0}^{\pm }\rangle$ is a degenerate state, which breaks the ${\hat{T}}_{x-y}$ symmetry. In contrast to previous studies^28^, the symmetry involved in our model does not contain an anti-unitary operator.

## Summary

In this paper we have studied the topological gapless state and edge modes of the Kitaev model on a square lattice. The advantage of studying the Kitaev model is that it is the minimal model in two dimensions where one can derive a number of analytical results for the topological gapless phase. It is shown that the topological gapless phase is characterized by two topological vortices with opposite chirality in the momentum plane. These two defects are unremovable until they get together some fixed points. We find that the two topological vortices do not meet when the system parameters drop in a large area of the parameter plane. Then it enhance the possibility to acquire the topological gapless phase in practice. Furthemore, we also analyze the symmetry which protects the topological gapless states the robustness under perturbations. In contrast to previous study, this symmetry does not involve any anti-unitary operation. This is may due to the fact that the magnetic flux is not necessary for the existence of the topological gapless phase in the present model.

In general, a quantum phase is a gapped phase, which exists in a region of parameter space. This region is very robust to parameter variations since the gap cannot close suddenly. Two different quantum phases are separated by gapless phase, which usually lives in the interface of two regions as quantum phase boundary. In this work, we find that the gapless phase also exists in a region of parameter space. And the region is also robust to parameter variations due to the topological nodual points, which cannot be removed suddenly. It indicates that, a transition from a gapped phase to a topological gapless phase should be regarded as a new type of topological quantum phase transition.

## Method

The essential nature of the topological boundary is that the band touching points are unavoidable under certain perturbation. We demonstrate this point by consider a perturbations with an extra diagonal hopping term. The perturbed Hamiltonian can be written as

35 $${H}_{{\rm{D}}}=H+{t}_{{\rm{D}}}\sum _{{\bf{r}}}({c}_{{\bf{r}}}^{\dagger }{c}_{{\bf{r}}+{\bf{b}}}+{\rm{h}}{\rm{.c}}{\rm{.}}),$$

where *t*_D_ denotes the diagonal hopping amplitude and vector **b** = *a* **i** + *a* **j**. Employing the Fourier transformations in Eq. (2), we still have

36 $${H}_{{\rm{D}}}=\sum _{{\bf{k}}}(\begin{array}{cc}{c}_{{\bf{k}}}, & {c}_{-{\bf{k}}}^{\dagger }\end{array}){h}_{{\bf{k}}}^{{\rm{D}}}(\begin{array}{c}{c}_{{\bf{k}}}^{\dagger }\\ {c}_{-{\bf{k}}}\end{array}),$$

where

37 $${h}_{{\bf{k}}}^{{\rm{D}}}={h}_{k}-{t}_{{\rm{D}}}\,\cos \,({k}_{x}+{k}_{y})(\begin{array}{cc}1 & 0\\ 0 & -1\end{array})\mathrm{.}$$

The spectrum is

38 $$\begin{array}{rcl}{\varepsilon }_{k}^{{\rm{D}}} & = & 2\{[\mu -t\,\cos \,{k}_{x}-t\,\cos \,{k}_{y}\\  &  & {+{t}_{{\rm{D}}}\cos ({k}_{x}+{k}_{y})]}^{2}+{{\rm{\Delta }}}^{2}{(\sin {k}_{x}+\sin {k}_{y})}^{2},\end{array}$$

which only has a shift on *t*, i.e., *μ* → *μ* + *t*_D_ cos (*k*_*x*_ + *k*_*y*_), from the spectrum *ε*_*k*_ in Eq. (7). Thus the zero point can be obtained directly as following. We are only interested in the non-trivial case with nonzero Δ. In this case, the positions of two vertices are determined by the equation

39 $${k}_{0x}=-{k}_{0y}=\pm \arccos (\frac{\mu +{t}_{{\rm{D}}}}{2t}).$$

The existence of the solution (*k*_0*x*_, *k*_0*y*_) requires that

40 $$0 < |\frac{\mu +{t}_{{\rm{D}}}}{t}| < 2.$$

**Figure 4:**
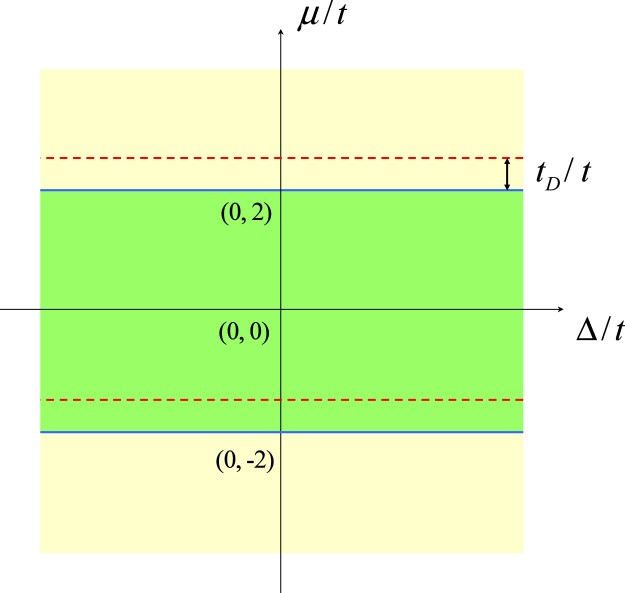
Phase diagram of the Kitaev model with *t*_D_− term on a square lattice on the parameter *μ* − Δ plane (in units of *t*). The blue lines indicate the original boundary, while the red ones denote the new boundary. It demonstrates that the topological gapless phase is robust under the *t*_D_− term perturbation.

It indicates that *t*_D_− term cannot destroy the topological gapless phase but just shifts the region along *μ*− axis by *t*_D_. Figure 4 illustrates this point. Then the gapless state is topologically invariant under the perturbation from the *t*_D_− term.
